# Supplementary material for: Digital descriptors sharpen classical descriptors, for improving genebank accession management: A case study on Arachis spp. and Phaseolus spp
Source: PLoS One. 2024 May 2;19(5):e0302158. doi: 10.1371/journal.pone.0302158 (PMC11065210; doi:10.1371/journal.pone.0302158)
Supplement: Attachment — Submitted filename: Review 21Ag23.docx [file pone.0302158.s003.docx]

Review:

Digital descriptors sharpen classical descriptors, improving the management of genebank accessions: A case study on Arachis spp. and Phaseolus spp.

The theme addressed by the manuscript (mss) is a comparison between descriptors used to characterise the phenotype of genebank accessions and phenomic evaluation of the same. The present study, provides a comparative study of phenomic descriptors and classical morphological descriptors, in two relatively distinct legume crop genera, largely native to the area of study.

Strategy adopted for the study is comprehensive- data collection is as per standardised methods; diverse species considered include- e.gs of closely related and distinct ones in Arachis and Phaseolus as well as hybrids in the latter; data has been gathered at more than one location.

Genebank management strategies are of interest to diverse users- it is essential that the work be presented such that it is understood by specialists in machine- based data collection and analysis as well as those studying germplasm conservation in the field and laboratory. The data collection and analysis presented by the authors is a good example of a preliminary study of the use of phenomics for determining variation and classification of genebank accessions. However, it is recommended that the mss be revised for proper understanding of the methods adopted and justification for using it; variation in descriptors that are effective in a species/ species complex, and so on; presently the reader has to delve in different portions of the mss to get the answers. All pertinent information is given in the mss but needs to be reorganised. Suggestions for modification of the mss are given under three heads- 1) Improvement of the ‘Material and Methods’; 2) Summing- up of results and discussing them- for legumes as a whole, the genera showing specific trends, diversity of the accessions studied, and probable reasons for the observed patterns; and 3) some examples in the text where corrections need to be made or further clarifications are required (the line numbers are marked for each item).

**Making the manuscript equally useful to diverse groups of workers handling germplasm diversity**:

Any study of variation in genebank accessions has to clearly enunciate methods and procedures adopted in the study that can be appreciated and understood by diverse users- on the one hand are the genebank workers familiar with handling the germplasm and characterising them using morphological/ numerical methods, and on the other, the machine- learning experts, developing and evaluating phenomic methods in various plant systems (viz. authors of the manuscript).

One way of achieving this would be by modifying the ‘material and methods’ portion- steps and procedures undertaken and justification for the same could be presented in the form of flowcharts and tabulations. This will not only increase clarity but will also reduce the descriptive portion; in its present form, methodology and specialised terminology, that are a part of machine- based data generation procedures, are included by the authors under introduction, results and some even in the discussion.

**An example of an aspect that has been to be consolidated:**

The results highlight the commonality of significant descriptors across the two distinct genera of legumes, an important point from the perspective of the stated aim- to integrate the characterization methodology into workflow of genebanks (line no.141)

The rationale behind selecting species for study should be given (supplementary data gives the list of species and accessions selected for study). Obviously good thought has gone into selection of the species/ species pairs in Arachis; the results convey the value of using AI for estimating variation represented in the genebank collections of such species pairs. Giving the rationale for selection under material and methods will aid in stating these points with more clarity in results and discussion. 145- 146; it has been mentioned in part in 248- 249. It will also make it easier, both for the reader to grasp the significance of the results and for the authors to highlight their results viz. trends in species belonging to the same section Erectoides of Arachis- more widespread species A. pintoi and the related wild species A. paraguariensis vis-à-vis the functional space; pinpointing redundancy 285- 291; distinguishing the comparative closely related species 379- 382..; determine the accessions per species that represent extreme functional descriptors, 485; etc.

**Mistakes/ needs more clarity**:

References quoted in parentheses by the authors, in some cases, do not convey the context in which the results obtained have been substantiated by past studies.; the reader needs to check out the original reference to know the relevance of the quoted reference. Other mistakes and queries too are listed below.

- Artificial intelligence (AI) is a set of tools for increasing our understanding many of life’s phenomena. Now AI has a real promise of delivering real value for humanity including in agriculture and food safety [18]. 86- 87: the description of AI’s promise and its relevance agriculture could be made more concise and focused.
- based on the fact of validating the phenotypic distances between species, in addition to being accessions that were in the process of seed regeneration. 154- 157: Needs to be clarified and better stated.
- Genebanks need to generate and apply procedures that identify accessions that display phenotypic variation, as well as to have phenotypic approaches to identify redundant accessions that are otherwise being duplicated. 227- 228 : streamline the sentence for genebanks’ need for identification and representation of phenotypic variation.
- a lower number of descriptors suitable for discriminating among forage peanut accessions compared to the classical descriptors. 286: use fewer descriptor instead of lower number
- In the classification there is a confusion especially among wild accessions of A. pintoi (18745, 18746, 288 1748, 22151, 22159, 22176). This shows again that the phenomic descriptors are accurately capturing the accessions redundancy. 288- 289: Reframe the explanation for confusion among accessions.
- The accessions of A. archeri (22226) and A. paraguariensis 290 (22636) present confusion indicating phenomic redundancy similarly to accessions of A. repens 291 (22162) and A. paraguariensis (22636) (Fig 3B). 290- 291: A. paraquariensis repeated.
- The combined descriptors (phenomic and classic) showed an intermediate area, however, there were 14 accessions evaluated (Fig 5E). 342- 343: ensure clarity in meaning of sentence.
- Not surprisingly, the phenomic descriptors show confusion especially in closely related accessions of beans and forage peanuts. However, interestingly, neither the classical descriptors nor the descriptor combination showed any confusion at all since all accessions were well distinguished. 429- 431: too verbose; it makes for heavy reading.
- The greater degree using the combination of descriptors (Fig 7G). 379: incomplete sentence
- However, a group of closely related accessions is clear due to the direction of the arrows, indicating similarity between them (Fig 7F). 385. Clarify.
- Germplasm explorations, the frame of reference was the variation between traits such as shape and color of leaves, flowers, and seeds, as well as morphological markers for new introductions in genebanks. 406- 407: Ambiguous; needs clarity.
- flower color (RHSwing and RHSstandart). 423: spelling of standard.
- The convex hull can be evaluated in two ways: The convex hull can be evaluated in two ways: (I) Vertices accessions could be considered core… 455- 456. Repetition.
- For example, functional space associates’ accessions of A. archieri, 459; correct name, A. archeri
- For example, functional space associates’ accessions of A. archieri, A. paraguariensis species that are closely related, as well as accessions of A. pintoi and A. repens. This is consistent with the classification of these two species under the section Caulorhizae [56]. 459- 461 : A. archeri and A. paraguariensis under section Erectoides- not mentioned.
- The evaluation of accessions of interest contemplating functional diversity analyses 543. Clarify use of contemplating.
- Our work demonstrates that by using a machine learning approach it is possible to find new digital descriptors and select only these classical descriptors that contribute mostly to the classification of a particular species or individual accessions. 528- 530. Examples from the data.
- Murgia, Tania Gioia, Debora Santo, Laura Nanni, Giovanna Attene, and Roberto Papa. "Beans (Phaseolus ssp.). 713- 714: correction, spp. for species to be used instead of ssp. which is the abbreviation for subspecies.
- Taxonomy, distribution, and ecology of the genus phaseolus (Leguminosae-papilionoideae) in .. 769: Phaseolus, Papilionoideae
- Fig 7: The two principal coordinate analysis components (PC1, PC2) of the functional specialization (FSpe), functional identity (FIde) and functional originality (FOri) indices. 961- 962: incomplete sentence.
- Title: strengthen instead of sharpen… for improved genebank accession management:….

**Conclusion**:

The manuscript serves as a valuable preliminary study in the field of phenomics, contributing to genebank management strategies. By improving the presentation and organization of the Material and Methods section, enhancing explanations of terms, and streamlining complex sentences, the manuscript can better contribute its findings to a diverse readership.
