# Supplementary material for: Digital descriptors sharpen classical descriptors, for improving genebank accession management: A case study on Arachis spp. and Phaseolus spp
Source: PLoS One. 2024 May 2;19(5):e0302158. doi: 10.1371/journal.pone.0302158 (PMC11065210; doi:10.1371/journal.pone.0302158)
Supplement: Attachment — Submitted filename: Comments.docx [file pone.0302158.s004.docx]

The manuscript “Digital descriptors sharpen classical descriptors, improving the management of genebank accessions: A case study on *Arachis* spp. and *Phaseolus* spp.” attractively written and study pertaining to the digital descriptors is new in the field of plant genetic resources for management of genebank accessions.

1. Abstract should be brief and precise elucidating the main content and results of the study
2. Author should include the total germplasm handling of the *Arachis* spp. and *Phaseolus* spp in the manuscript available in the genebank.
3. Instead of table, a good quality global map can represent the diverse accessions clearly.
4. There is no discussion about the statistical experimental design for the study.
5. There is vast variability in qualitative traits in *Arachis* and *Phaseolus* spp., author include only 5 species, one is unknown (16 accessions) in case of *Arachis* and 6 species including two Hybrids (14 accessions) in case of *Phaseolus.* There should be inclusion of more diverse accessions and species (cultivated; *Arachis hypogeal* and *Phaseolus vulgaris*) in the study for better representation of qualitative traits to prepare digital descriptors.
6. Sentence in line 169 is not complete.
7. Author is suggested to prepare an informative flow line diagram for the material and methods for better understanding of methodology followed in the study.

The manuscript needs additional information on the aspects cited above, therefore, requires a "Minor revision" for publication of the manuscript in PlosOne to keep up the high reputation of the journal.
