# Supplementary material for: Digital descriptors sharpen classical descriptors, for improving genebank accession management: A case study on Arachis spp. and Phaseolus spp
Source: PLoS One. 2024 May 2;19(5):e0302158. doi: 10.1371/journal.pone.0302158 (PMC11065210; doi:10.1371/journal.pone.0302158)
Supplement: Attachment — Submitted filename: Response to ReviewersR.docx [file pone.0302158.s005.docx]

February 16 , 2024

Kuldeep Tripathi

Academic Editor

PLOS ONE

Dear Kuldeep Tripathi we want to inform you that all the corrections suggested by the 2 reviewers to our manuscript entitled “Digital descriptors sharpen classical descriptors, improving the management of genebank accessions: A case study on Arachis spp and Phaseolus spp.” with the identification code: PONE-D-23-21318, were successfully incorporated and a new version of the manuscript was created. In my name and also of all coauthors we think that this version could be considered for publication in the journal PLOS ONE.

Based on the reviewer’s comments and their suggestions, we have made substantial modifications to the original manuscript and proofread it carefully. We are grateful to the reviewers, as their comments were helpful to improve the manuscript quality, increased readability and flow. We have included a better description especially of the methodological details. Please, see the changes incorporated in the track changes format. We hope that in your eyes the manuscript is highly improved, and you again will consider our manuscript for publication in the journal. Additionally, the language of the manuscript was checked again by a native English speaker.

Please, see detailed responses to the reviewers comments. If you still hesitate on our responses or that something lacks some significant idea, please, do not hesitate to contact us again.

With all respect

Diego Conejo, Milan O. Urban

**Reviewer #1:**

**General comments**

The theme addressed by the manuscript (mss) is a comparison between descriptors used to characterise the phenotype of genebank accessions and phenomic evaluation of the same. The present study, provides a comparative study of phenomic descriptors and classical morphological descriptors, in two relatively distinct legume crop genera, largely native to the area of study.

Strategy adopted for the study is comprehensive- data collection is as per standardised methods; diverse species considered include- e.gs of closely related and distinct ones in Arachis and Phaseolus as well as hybrids in the latter; data has been gathered at more than one location.

Genebank management strategies are of interest to diverse users- it is essential that the work be presented such that it is understood by specialists in machine- based data collection and analysis as well as those studying germplasm conservation in the field and laboratory. The data collection and analysis presented by the authors is a good example of a preliminary study of the use of phenomics for determining variation and classification of genebank accessions. However, it is recommended that the mss be revised for proper understanding of the methods adopted and justification for using it; variation in descriptors that are effective in a species/ species complex, and so on; presently the reader has to delve in different portions of the mss to get the answers. All pertinent information is given in the mss but needs to be reorganized. Suggestions for modification of the mss are given under three heads- 1) Improvement of the ‘Material and Methods’; 2) Summing- up of results and discussing them- for legumes as a whole, the genera showing specific trends, diversity of the accessions studied, and probable reasons for the observed patterns; and 3) some examples in the text where corrections need to be made or further clarifications are required (the line numbers are marked for each item).

**R: The above mentioned sections were significantly changed. Please see details in the track changes or in the new version of the ms. Thank you for your important views and suggestions. We believe these helped ms to be more readable and thoughts acceptable.**

Any study of variation in genebank accessions has to clearly enunciate methods and procedures adopted in the study that can be appreciated and understood by diverse users- on the one hand are the genebank workers familiar with handling the germplasm and characterising them using morphological/ numerical methods, and on the other, the machine- learning experts, developing and evaluating phenomic methods in various plant systems (viz. authors of the manuscript).

One way of achieving this would be by modifying the ‘material and methods’ portion- steps and procedures undertaken and justification for the same could be presented in the form of flowcharts and tabulations. This will not only increase clarity but will also reduce the descriptive portion; in its present form, methodology and specialised terminology, that are a part of machine- based data generation procedures, are included by the authors under introduction, results and some even in the discussion.

**R: we have added a new workflow scheme which hopefully increased clarity of the proposed methodology. The MaMs were also modified and some important details were added. Please, see track changes or new original document.**

**An example of an aspect that has been to be consolidated:**

The results highlight the commonality of significant descriptors across the two distinct genera of legumes, an important point from the perspective of the stated aim- to integrate the characterization methodology into workflow of genebanks (line no.141)

The rationale behind selecting species for study should be given (supplementary data gives the list of species and accessions selected for study). Obviously good thought has gone into selection of the species/ species pairs in Arachis; the results convey the value of using AI for estimating variation represented in the genebank collections of such species pairs. Giving the rationale for selection under material and methods will aid in stating these points with more clarity in results and discussion. 145- 146; it has been mentioned in part in 248- 249. It will also make it easier, both for the reader to grasp the significance of the results and for the authors to highlight their results viz. trends in species belonging to the same section Erectoides of Arachis- more widespread species A. pintoi and the related wild species A. paraguariensis vis-à-vis the functional space; pinpointing redundancy 285- 291; distinguishing the comparative closely related species 379- 382..; determine the accessions per species that represent extreme functional descriptors, 485; etc.

R: we have added the rationale behind the accession selection into lines 157-167

**Mistakes/ needs more clarity**:

References quoted in parentheses by the authors, in some cases, do not convey the context in which the results obtained have been substantiated by past studies.; the reader needs to check out the original reference to know the relevance of the quoted reference.

**Specific comments**

1. Artificial intelligence (AI) is a set of tools for increasing our understanding many of life’s phenomena. Now AI has a real promise of delivering real value for humanity including in agriculture and food safety [18]. 86- 87: the description of AI’s promise and its relevance agriculture could be made more concise and focused.

R: Thank you for your suggestion. We modified lines regarding AI description. Please see lines 67-70 and on.

2. based on the fact of validating the phenotypic distances between species, in addition to being accessions that were in the process of seed regeneration. 154- 157: Needs to be clarified and better stated.

R: Thank you for your suggestion. We modified lines in accession selection in the part of Classical descriptors, lines 168 and on.

3. Genebanks need to generate and apply procedures that identify accessions that display phenotypic variation, as well as to have phenotypic approaches to identify redundant accessions that are otherwise being duplicated. 227- 228 : streamline the sentence for genebanks’ need for identification and representation of phenotypic variation.

R: Thank you for your suggestion. We modified the lines mentioned. Please see the changes starting from line 235.

4. a lower number of descriptors suitable for discriminating among forage peanut accessions compared to the classical descriptors. 286: use fewer descriptor instead of lower number

R: The part was modified according to your suggestion, thanks!

5. In the classification there is a confusion especially among wild accessions of A. pintoi (18745, 18746, 288 1748, 22151, 22159, 22176). This shows again that the phenomic descriptors are accurately capturing the accessions redundancy. 288- 289: Reframe the explanation for confusion among accessions.

R: The part was modified according to your suggestion, thanks! “In the confusion matrix of phenomic descriptors, *A. pintoi'*s wild accessions (18745, 18746, 1748, 22151, 22159, and 22176) had the highest level of similarity. This indicates that the digital descriptors accurately capture the phenotypic redundancy of those accessions where classical descriptors do not show differences”.

6. The accessions of A. archeri (22226) and A. paraguariensis 290 (22636) present confusion indicating phenomic redundancy similarly to accessions of A. repens 291 (22162) and A. paraguariensis (22636) (Fig 3B). 290- 291: A. paraquariensis repeated.

R: The part was modified according to your suggestion, thanks! “For example, the *A. paraguariensis* (22636) presents similarity with the *A. archeri* (22226) and *A. repens* (22162) indicating phenotypic closeness to these accessions(Fig 4B).”

7. The combined descriptors (phenomic and classic) showed an intermediate area, however, there were 14 accessions evaluated (Fig 5E). 342- 343: ensure clarity in meaning of sentence.

R: The part was modified according to your suggestion, thanks!

8. Not surprisingly, the phenomic descriptors show confusion especially in closely related accessions of beans and forage peanuts. However, interestingly, neither the classical descriptors nor the descriptor combination showed any confusion at all since all accessions were well distinguished. 429- 431: too verbose; it makes for heavy reading.

R: Thank you for this comment. We have reformulated the statement starting from line 437 to: The phenomic descriptors logically reveal some uncertainty in accessions determination particularly in closely related accessions of forage peanuts and beans. This is not a surprise and will likely change with the newly generated knowledge. However, since all accessions were clearly differentiated, neither the classical descriptors nor the descriptor combination displayed any uncertainty at all.

9. The greater degree using the combination of descriptors (Fig 7G). 379: incomplete sentence

R: The part was modified according to your suggestion, starting from 388. “The FSpe analysis showed the phenomic (Fig 8A) and classical descriptors (Fig 8D) discriminated A. pintoi against *A. paraguariensis*. Many *A. pintoi* accessions are clustered using the classical and phenomic descriptors, yet to a greater degree in the combination (Fig 8G).”

10. However, a group of closely related accessions is clear due to the direction of the arrows, indicating similarity between them (Fig 7F). 385. Clarify.

R: reformulated to “The results showed that the combination of classical and phenomic descriptors can discriminate between forage peanuts and between bean accessions more accurately than using them independently. For both bean and peanut forage accessions the first morphospaces of seed and leaf (Fig 3A) are important to discriminate accessions. “

11. Germplasm explorations, the frame of reference was the variation between traits such as shape and color of leaves, flowers, and seeds, as well as morphological markers for new introductions in genebanks. 406- 407: Ambiguous; needs clarity.

R: The part was modified according to your suggestion, thanks! The new text is: “Historically, the variability in morphological and colorimetric traits in leaves, flowers, and seeds was a key factor in determining the genetic representativeness of the collected accessions during the germplasm explorations. List of standardized descriptors relating morphological and agronomic parameters served as a starting point for germplasm introductions into different agro-environments [46, 47]”.

12. flower color (RHSwing and RHSstandart). 423: spelling of standard.

R: Thank you for finding this typo. We have edited the standart to “standard.”

13. The convex hull can be evaluated in two ways: The convex hull can be evaluated in two ways: (I) Vertices accessions could be considered core… 455- 456. Repetition.

R: Thanks to find this duplication. We have eliminated the first part of the status.

“The convex hull can be evaluated in two ways: (I) Vertices accessions could be considered a core or functional collections; (II) Determining redundant accessions allows genomic evaluations to be performed on a smaller number of accessions and verify their similarity. For example, functional space associates *A. archeri* and *A. paraguariensis* that are closely related to section Erectoides. Also the accessions of *A. pintoi* and *A. repens* under the section Caulorhizae are well associated [56]”.

14. For example, functional space associates’ accessions of A. archieri, 459; correct name, A. archeri

R: Thank you for finding this brutal typo. We have edited the correct latin name.

15. For example, functional space associates’ accessions of A. archieri, A. paraguariensis species that are closely related, as well as accessions of A. pintoi and A. repens. This is consistent with the classification of these two species under the section Caulorhizae [56]. 459- 461 : A. archeri and A. paraguariensis under section Erectoides- not mentioned.

R: Thanks. Please, see the response to question 13.

16. The evaluation of accessions of interest contemplating functional diversity analyses 543. Clarify use of contemplating.

R: We have reformulated the statement: The evaluation of accession of interest considers and reflects the functional diversity analyses as well.

17. Our work demonstrates that by using a machine learning approach it is possible to find new digital descriptors and select only these classical descriptors that contribute mostly to the classification of a particular species or individual accessions. 528- 530. Examples from the data.

R:Based on your suggestion, the original text was changed to: ”Our work proposes a methodological route that allows the integration of high-throughput phenotyping, artificial intelligence and functional diversity indices that allow exploring the dimensionality of the phenome in genebanks. The study of the phenome in germplasm collections can contribute to the selection of accessions via functional traits with potential for crop improvement and to understand the evolutionary aspects that conditioned the phenotypic diversity of plant genetic resources”.

18. Murgia, Tania Gioia, Debora Santo, Laura Nanni, Giovanna Attene, and Roberto Papa. "Beans (Phaseolus ssp.). 713- 714: correction, spp. for species to be used instead of ssp. which is the abbreviation for subspecies.

R: Yes, done

19. Taxonomy, distribution, and ecology of the genus phaseolus (Leguminosae-papilionoideae) in .. 769: Phaseolus, Papilionoideae

R: Thank you for this question, however, this is an official version of the reference which cannot be changed as was published this way.

20. Fig 7: The two principal coordinate analysis components (PC1, PC2) of the functional specialization (FSpe), functional identity (FIde) and functional originality (FOri) indices. 961- 962: incomplete sentence.

R: Please find a new legend in Figure 8 with corrected sentences.

21. Title: strengthen instead of sharpen… for improved genebank accession management:….

R: Thank you very much for this suggestion. However, the title in this version was suggested by native speaker and official English language editor. I hope it is ok if we follow the original version.

**Conclusion**:

The manuscript serves as a valuable preliminary study in the field of phenomics, contributing to genebank management strategies. By improving the presentation and organization of the Material and Methods section, enhancing explanations of terms, and streamlining complex sentences, the manuscript can better contribute its findings to a diverse readership.

**R: Dear anonymous reviewer, your suggestions and ideas were almost all accepted very well and surely increased the flow of the text. We realized writing about these new technologies is not easy and also to find stakeholders for this text will not be easy. We hope you will like changes we did on your suggestions, however, some parts were totally remodeled. If doubts or questions, please compare the original and track changes/new version.**

**Reviewer #2:**

**General comments**

The manuscript “Digital descriptors sharpen classical descriptors, improving the management of genebank accessions: A case study on *Arachis* spp. and *Phaseolus* spp.” attractively written and study pertaining to the digital descriptors is new in the field of plant genetic resources for management of genebank accessions.

**Specific comments**

1. Abstract should be brief and precise elucidating the main content and results of the study

R: Dear Reviewer, thank you for your comment. We significantly reduced the abstract. Please see the new version or track changes version.

2. Author should include the total germplasm handling of the *Arachis* spp. and *Phaseolus* spp in the manuscript available in the genebank.

R: The list of the materials is in Table 1. The explanation and justification is in the response below. The question on experimental design and selected germplasm is normally crucial when normal experiment is prepared. Please, understand, based on the below explanation, that the accessions were selected based on the below 3 points, which means accessions available at the time of routine regeneration processes.

3. Instead of table, a good quality global map can represent the diverse accessions clearly.

R: As explained below according to three points, accessions for this study were not selected based on the theri ecogeographic distribution but immediate availability during the routine regeneration processes in CIAT genebank. All experiments followed well established and rigorous SOP. “The sample size follows the classical morphological characterization methods [5 - 7] and three important criteria were used to select accessions for this study: (1) The seed regeneration process: In Tenerife and Palmira, a mapping group of accessions were sown to produce seeds. (2) The present species diversity: In order to support the process of distinguishing and classifying species, we considered the diversity of Phaseolus and Arachis species as well as interspecific hybrids that were present during the particular seed regeneration process. (3) The phenotypic variation: Within a species, accessions were chosen based on morphological, colorimetric variation and seed availability. Since phenotypic characterisation of accessions was fully complementary during the routine seed regeneration process, our study could not change or take an experimental design into consideration”.

4. There is no discussion about the statistical experimental design for the study.

R: Please, find the explanation above in the answer to the questions 2 and 3.

5. There is vast variability in qualitative traits in *Arachis* and *Phaseolus* spp., author include only 5 species, one is unknown (16 accessions) in case of *Arachis* and 6 species including two Hybrids (14 accessions) in case of *Phaseolus.* There should be inclusion of more diverse accessions and species (cultivated; *Arachis hypogeal* and *Phaseolus vulgaris*) in the study for better representation of qualitative traits to prepare digital descriptors.

R: This is again a very important comment and normally will be crucial for similar analysis and understanding of the results. However, based on the q 2-4, you can see the goal of this study is actually not to include the highest variability possible but to follow the routine regeneration and create methodologically correct workflow useful for CIAT genebank. Furthermore, the proposed workflow is practical and reflect the reality of CIAT genebank where regeneration of accessions do not depend on their origin but needs. In that sense, our methodology counts with the fact that it will serve as a complementary to routine SOPs. We are sure that the results and again the goal are sufficient proof that even with reduced genetic variability this was satisfactorily accomplished.

6. Sentence in line 169 is not complete.

R: Yes, the line was completed as suggested.

7. Author is suggested to prepare an informative flow line diagram for the material and methods for better understanding of methodology followed in the study.

R: The workflow (hopefully you will like it) was included as suggested. Please see figures 1 and 9.

Sincerely, on behalf of all authors,

Diego Felipe Conejo and Milan Urban
